# The *CYP51F1* Gene of *Leptographium qinlingensis*: Sequence Characteristic, Phylogeny and Transcript Levels

**DOI:** 10.3390/ijms160612014

**Published:** 2015-05-26

**Authors:** Lulu Dai, Zhumei Li, Jiamin Yu, Mingyuan Ma, Ranran Zhang, Hui Chen, Thanh Pham

**Affiliations:** College of Forestry, Northwest A&F University, Yangling 712100, China; E-Mails: dailulu@nwafu.edu.cn (L.D.); zhumeili@nwafu.edu.cn (Z.L.); yujiam@nwafu.edu.cn (J.Y.); mingyuanma@nwafu.edu.cn (M.M.); ranran@nwafu.edu.cn (R.Z.); efphamthanh@gmail.com (T.P.)

**Keywords:** *Leptographium qinlingensis*, lanosterol 14-α demethylase, terpenoid, homology modeling, molecule docking

## Abstract

*Leptographium qinlingensis* is a fungal associate of the Chinese white pine beetle (*Dendroctonus armandi*) and a pathogen of the Chinese white pine (*Pinus armandi*) that must overcome the terpenoid oleoresin defenses of host trees. *L. qinlingensis* responds to monoterpene flow with abundant mechanisms that include export and the use of these compounds as a carbon source. As one of the fungal cytochrome P450 proteins (CYPs), which play important roles in general metabolism, CYP51 (lanosterol 14-α demethylase) can catalyze the biosynthesis of ergosterol and is a target for antifungal drug. We have identified an *L. qinlingensis* CYP51F1 gene, and the phylogenetic analysis shows the highest homology with the 14-α-demethylase sequence from *Grosmannia clavigera* (a fungal associate of *Dendroctonus ponderosae*). The transcription level of CYP51F1 following treatment with terpenes and pine phloem extracts was upregulated, while using monoterpenes as the only carbon source led to the downregulation of CYP5F1 expression. The homology modeling structure of CYP51F1 is similar to the structure of the lanosterol 14-α demethylase protein of *Saccharomyces cerevisiae* YJM789, which has an *N*-terminal membrane helix 1 (MH1) and transmembrane helix 1 (TMH1). The minimal inhibitory concentrations (MIC) of terpenoid and azole fungicides (itraconazole (ITC)) and the docking of terpenoid molecules, lanosterol and ITC in the protein structure suggested that CYP51F1 may be inhibited by terpenoid molecules by competitive binding with azole fungicides.

## 1. Introduction

“Bark beetles that colonize living conifers are frequently associated with specific fungi that are carried in specialized structures or on the body surface” [1]. The conclusion repeated in the literature suggests that pathogenic blue-stain fungi are primarily responsible or required for the mortality of trees attacked by bark beetles followed a logical thread, beginning with the observations that the sapwood of beetle-killed trees is stained [1,2,3]. *Leptographium* sp. associated with the Chinese white pine beetle (*Dendroctonus armandi*) helps the bark beetles to overcome the resistance system of the host trees [4]. *Leptographium qinlingensis* is an active participant in the death of the Chinese white pine (*Pinus armandi*) on Qinling Mountain of China [5,6]. Symbiotic fungi destroyed the bleeding cells, blocked up resin canals of the host trees and killed epithelial cells, resulting in disorders of the nutrient and water metabolisms of the host and the death of host trees [7,8]. The three toxins (6-methoxymethyleugenin, maculosin and cerevisterol) synthesized by *L. qinlingensis* are phytotoxic to *P. armandi* seedlings [9]. In addition, inoculation with *L. qinlingensis* increases the concentrations of monoterpenes and sesquiterpenes in the phloem and xylem of the *P. armandi* seedlings [10].

The *Grosmannia clavigera* vectored by *Dendroctonus ponderosae* has caused a rapid, large-scale decline of *Pinus contorta* in western North America [11]. Lodgepole pine also dies when inoculated at a high density of pathogenic fungus, like *Leptographium longiclavatum*, without the beetles [12]. The genome and transcriptome of *G. clavigera* induced by exposure to lodgepole pine phloem extract (LPPE) or oleoresin terpenoids were reported by DiGuistini [13]. The comparative analyses of the expression profiles of *G. clavigera* grown on monoterpenes, triglycerides or oleic acid showed that cytochrome P450 (CYP) may be involved in the utilization of triglycerides, oleic acid and monoterpenes as a carbon source and that the CYPs may detoxify pine defense compounds [14].

Fungal lanosterol 14-α demethylase (CYP51) is the key enzyme in the ergosterol biosynthesis pathway and a major antifungal drug target [15,16]. In contrast to most other CYP families, lanosterol 14-α demethylase has a preserved narrow function involving the removal of the 14-methyl group of sterol precursors and has retained high substrate specificity throughout evolution [17]. However, only five structurally similar 14-α-methylsterol substrates are known, including lanosterol, 24,25-dihydrolanosterol, 24-methylenedihydrolanosterol, obtusifoliol and 4-β-desmethyllanosterol [18]. Azoles inhibit CYP51, causing the accumulation of methylated sterols, the depletion of ergosterol and the inhibition of cell growth [19], and epoxiconazole and itraconazole (ITC) are azole fungicides that inhibit the fungal CYP51 [20,21].

There are many similarities between *L. qinlingensis* and *G. clavigera*, as they are associated fungi of *Dendroctonus* beetles. Here, we identified a CYP51 gene from *L. qinlingensis* and compared its homology with other fungal CYP51s, including the lanosterol 14-α demethylase sequence from *G. clavigera*. The CYP expression profiles of *G. clavigera* grown on monoterpenes, triglycerides or oleic acid were identified, but did not include CYP51 [14]. The expression profiles of CYP51F1 in *L. qinlingensis* mycelia grown on monoterpenes, oleic acid, oleoresin terpenoids and Chinese white pine phloem methanol extract (CWPPE) were significantly influenced by terpenoids. A cytochrome P450 homology model for CYP51F1 has been constructed for molecule docking to understand the interaction between the CYP51F1 protein and its ligands (terpenoid, lanosterol and itraconazole).

## 2. Results

### 2.1. Identification of the Cytochrome P450 Gene

**Figure 1:**
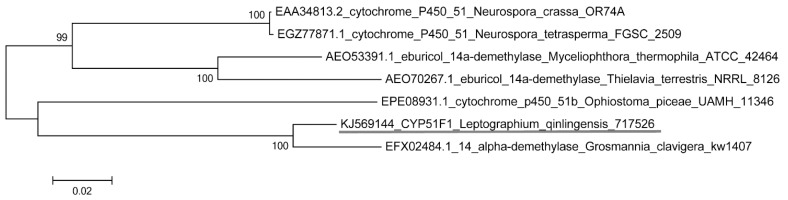
A maximum likelihood tree of cytochrome P450 gene from *L. qinlingensis* with partial sequences was performed using the amino acidic substitution model WAG model with a Gamma (−lnL = 2883.91). The CYP51F1 from *L. qinlingensis* is shown with an underline. The values of the bootstrap after 500 pseudoreplicates are shown at the nodes.

The CYP gene set of the CYP51 family, which had bootstrap values >99%, was found by Maximun Likelihood phylogenetic (ML-phylogenetic) analysis of the putative full-length amino acid sequences (Figure 1). BLAST searches indicated that CYP genes expressed in *L. qinlingensis* were similar to members of the gene family CYP51 reported in other species (Table 1). The full-length sequence from the CYP51 gene shared the highest level of amino acid sequence identity with variants from the fungal species *G. clavigera* kw1407, *N. crassa* OR74A, *N. tetrasperma* FGSC 2509, *M. thermophila* ATCC 42464, *T. terrestris* NRRL 8126 and *O. piceae* UAMH 11346 (Table 1). Amino acid sequence identity between partial-length sequences within each gene ranged from 86.3%–96.9% with respect to matched GenBank sequences (inter-variant). The sequence identity between the full length sequence of each gene and GenBank reference sequences was 77.5%–91.1%. The lanosterol 14-α demethylase sequence from *G. clavigera* kw1407 had the highest sequence identity.

### 2.2. Physicochemical Properties and Bioinformatics Analysis

**Figure 2:**
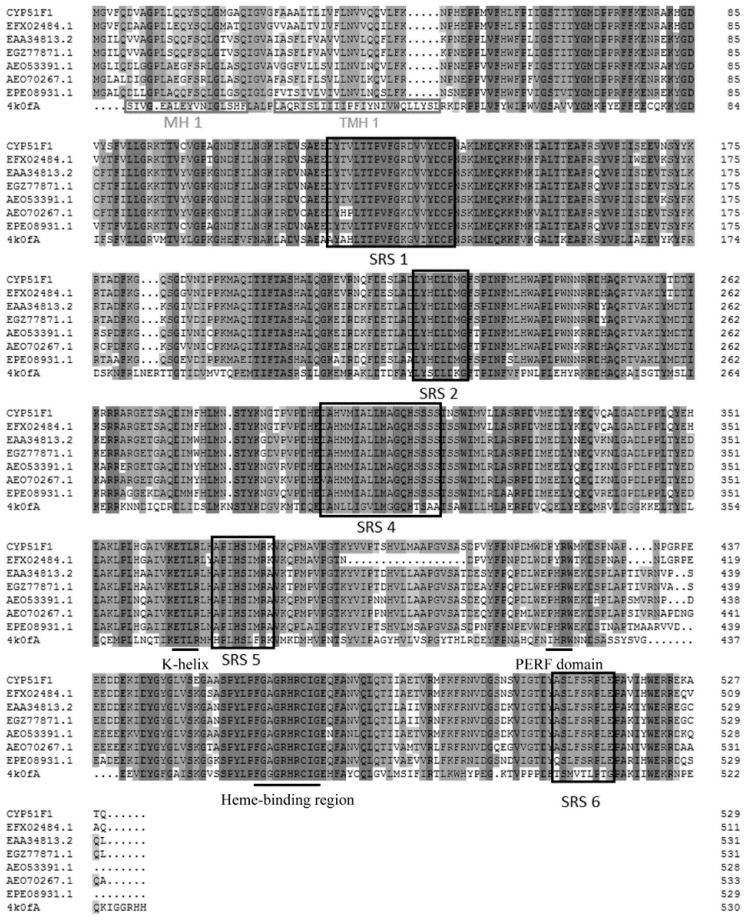
Multiple sequence alignment. The alignment included CYP51F1 from *L. qinlingensis*, the *Saccharomyces cerevisiae* YJM789 lanosterol 14-α demethylase protein (PDB code: 4k0f chain A) sequence and the genes used in Figure 1. The heme-binding region (FXXGXRXCXG), PERF domain (PXRX) and K-helix (EXXR) are shown with an underline. Substrate recognition sites (SRSs) 1–2 and 4–6 were manually determined according to 4k0f-A. The predicted membrane interactions of 4k0f-A (MH1 and TMH1) are in grey boxes.

The full-length sequences of CYP51F1 (named by the P450 nomenclature committee) gene was 1996 bp with a 1590-bp open reading frame (ORF), which encoded 529 amino acids (Table 2). The CYP51F1 gene was flanked by 5ʹ and 3ʹ untranslated regions (UTRs) varying in length from 206–200 bp. The predicted molecular mass was 59.31 kDa with an isoelectric point of 6.82 (Table 2). The predicted subcellular localization of the P450 protein revealed a putative code for a typical membrane protein containing approximately 20 hydrophobic residues that are likely membrane anchors in the endoplasmic reticulum (Table 2). The alignment and comparison of the deduced amino acid sequence of CYP51F1 from *L. qinlingensis* with the *S. cerevisiae* YJM789 lanosterol 14-α demethylase protein sequence allowed for the identification of the substrate recognition sites (Figure 2).

**Table 1:** Aminoacidic identity of putative cytochrome P450 cDNA isolated from *L. qinlingensis* with cytochromes P450 sequences in fungus.

*L. qinlingensis* P450 Name	Blast Matches in Gene Bank			Identity % in Partial and Full Length ^a^		Identity % in Full Length ^b^
	Species	P450 Name	Accession Number	A	B	BlastP
CYP51F1	*Grosmannia clavigera* kw1407	14 alpha-demethylase	EFX02484.1	96.9	91.1	91
	*Neurospora crassa* OR74A	cytochrome P450 51	EAA34813.2	88.2	81.2	82
	*Neurospora tetrasperma* FGSC 2509	cytochrome P450 51	EGZ77871.1	88.2	81.0	82
	*Myceliophthora thermophila* ATCC 42464	eburicol 14a-demethylase	AEO53391.1	87.6	79.8	80
	*Thielavia terrestris* NRRL 8126	eburicol 14a-demethylase	AEO70267.1	86.3	77.5	77
	*Ophiostoma piceae* UAMH 11346	cytochrome p450 51b	EPE08931.1	87.0	80.4	81

A = identity percentage among partial sequences of all different genes; B = identity percentage among complete sequences of the same gene of different species; ^a^ As predicted by MATGAT [22]; ^b^ As predicted by BLAST (www.ncbi.nlm.nih.gov) [23].

**Table 2:** Physicochemical properties and cellular localization of putative *L. qinlingensis* P450 protein.

P450 Name	Accession No.	Full Length	ORF Size (aa/bp) ^a^	Flanked (bp)	*M*w (kDa) ^a^	I.P. ^a^	Signal Peptide Prediction ^b^
CYP51F1	KJ569144	1996 bp	529 aa 1590 bp	206–200	59.31	6.82	SP 0.554 mTP 0.048 other 0.625

^a^ As predicted by the ProtParam program [24]; ^b^ As predicted by Target P 1.1 [25]; *M*w: molecular weight; I.P.: isoelectric point; SP: secretory pathway; mTP: mitochondrial targeting peptide.

### 2.3. RT-qPCR

**Figure 3:**
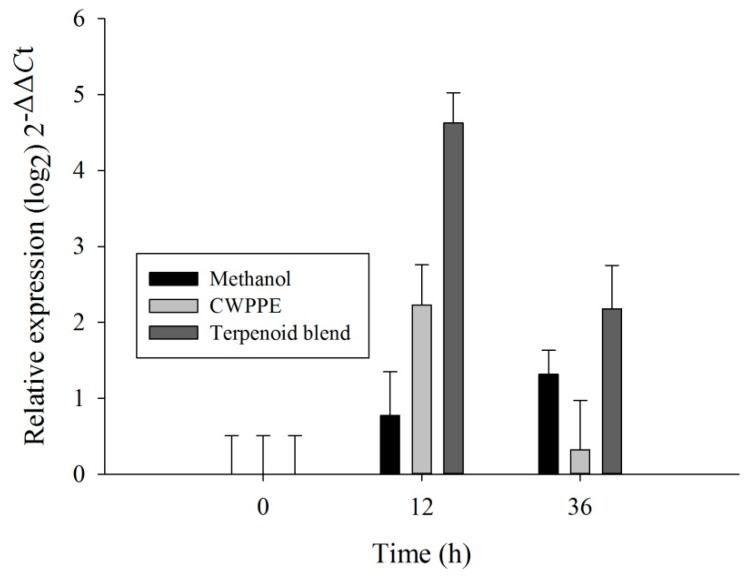
The quantitative expression of the CYP51 gene (mean ± SE) in mycelia grown on CM (complete medium) + methanol and CW (Chinese white pine phloem methanol extract (CWPPE)) and T (terpenoid blend) of *L. qinlingensis*. CYP expression was normalized with respect to EF1. The 2^−ΔΔ*C*t^ and SE values were transformed at log_2_ for plotting.

To determine if *L. qinlingensis* CYP51F1 had a possible role in the detoxification of pine defense chemicals, we analyzed the expression profiles of CYP51F1 from mycelia grown on complete medium treated with a terpenoid blend (CM + T) or with Chinese white pine phloem methanol extract (CWPPE; CM + CW) for 12 and 36 h. Statistically-significant differences were found among treatments and time (one-way ANOVA, treatments: *F* = 54.536, *df* = 2, *p* < 0.001; time: *F* = 25.399, *df* = 2, *p* < 0.001). CYP51F1 was upregulated after being exposed to the terpene blend for 12 and 36 h, and at 36 h, the transcription level was lower than at 12 h (Figure 3). At 12 h following CWPPE treatment (CM + CW, 12 h), the expression of CYP51F1 was significantly affected compared to the methanol treatments; however, one day later (CM + CW, 36 h), the expression of the CYP51F1 gene was significantly downregulated (Figure 3).

**Figure 4:**
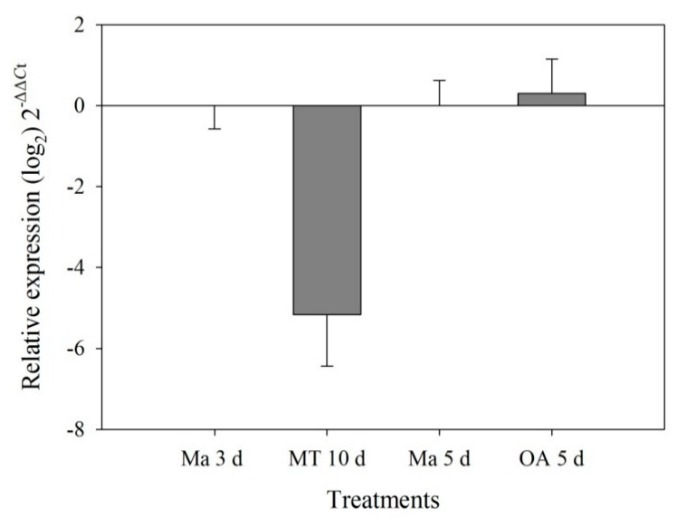
Quantitative expression of the CYP51 gene (mean ± SE) in *L. qinlingensis* grown on YNB (yeast nitrogen base without amino acids) + Ma (mannose) and MT (monoterpenes) and OA (oleic acid) with different carbon sources. CYP expression was normalized with respect to EF1. The 2^−ΔΔ*C*t^ and SE values were transformed at log_2_ for plotting.

To determine whether CYP51F1 was involved in the utilization of different carbon sources, we analyzed CYP gene expression profiles of *L. qinlingensis* grown on minimal medium with a single carbon source: a monoterpene blend for 10 days (yeast nitrogen base (YNB) + MT) and long-chain fatty acids (oleic acid; YNB + OA) for five days. A statistically-significant difference was found only between YNB + MT (10 days) and YNB + Ma (mannose) (3 days) (one-way ANOVA, *F* = 41.181, *df* = 1, *p* = 0.003). In mycelia grown on monoterpenes as the sole carbon source (YNB + MT), CYP51F1 was significantly downregulated (Figure 4). The expression of CYP51F1 displayed almost no change between YNB + OA (five days) and YNB + Ma (five days) (one-way ANOVA, *F* = 0.249, *df* = 1, *p* = 0.644) (Figure 4).

### 2.4. Modeling Structure of CYP51F1 and Molecule Docking

There were 50 protein sequences used as CYP51F1’s templates, from which we selected four proteins as representative sequences, based on the highest identity and sequence coverage with the available template structures. The protein sequence of CYP51F1 shared 44.29% and 39.77% identity with the four template structures (Table 3). The selected structures of CYP51F1 were then verified using various scoring methods. The QMEAN4, VERIFY_3D score, ERRAT score and the Ramachandran plot score of the best protein model were −4.67, 86.02%, 78.125% and 87.0%, respectively (Table 3).

**Table 3:** Verification of four modeled protein structure from the SWISS-MODEL program.

Model	Template	Identify %	QMEAN4 ^a^	ERRAT ^b^	VERIFY_3D ^b^ %	PROCHECK ^b^ %
1	4k0f.1 A	44.29	−4.67	78.125	86.02	87.0
2	4lxj.1 A	44.29	−5.15	77.193	84.29	88.4
3	3ld6.1 B	39.77	−7.74	61.290	87.34	85.7
4	3juv.1 A	39.77	−7.62	54.310	87.97	85.7

^a^ As a verification value from the SWISS-MODEL program [26]; ^b^ As the verification program from Structural Analysis and Verification Server (SAVES) (http://nihserver.mbi.ucla.edu/SAVES/).

**Figure 5:**
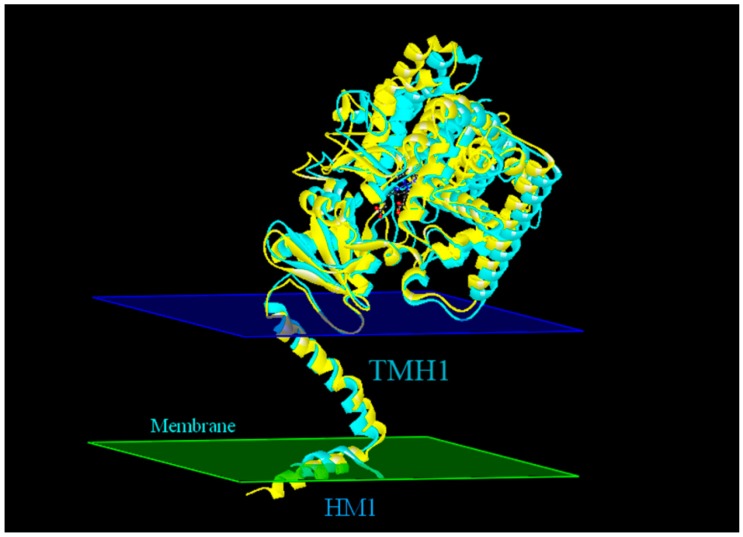
Superimposition of the model protein CYP51F1 (cyan) and template protein 4k0f A (yellow). The superimposed structure of the protein CYP51F1 with the template protein 4k0f chain A demonstrates an RMSD (Root-mean-square deviation) value of 10.805 Å. The predicted membrane interactions of 4k0f chain A (MH1 and TMH1) were added as two slices (cytoplasmic membrane in blue and luminal membrane in green).

**Figure 6:**
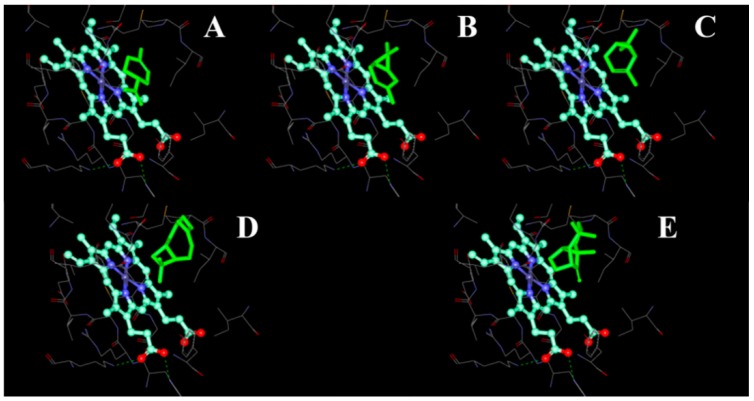
The binding pocket of CYP51F1 with terpenoid molecules. The terpenoid molecules limonene (**A**); carene (**B**); pinene (**C**); β-caryophyllene (**D**) and longifolene (**E**) are drawn as a green stick representation. The heme-porphyrin is drawn as a ball and stick representation with carbon atoms in cyan.

We determined the fold conservation of our generated models. The superposed structures of CYP51F1 upon 4k0f.1 chain A resulted in a Root-mean-square deviation (RMSD) value of 10.805 Å (Figure 5). The 50 *N*-terminal amino acids revealed two helices oriented at approximately 60° to each other (Figure 6). The *N*-terminal helix (membrane helix 1 (MH1), residues 8–14) is amphipathic and has extensive crystal contacts with symmetry-related molecules (Figure 5). MH1 connects to TMH1 via a short turn (residues 15–23), a slightly kinked helix 36.99 Å long (residues 24–47) that is of sufficient length to traverse the lipid bilayer (Figure 5).

**Figure 7:**
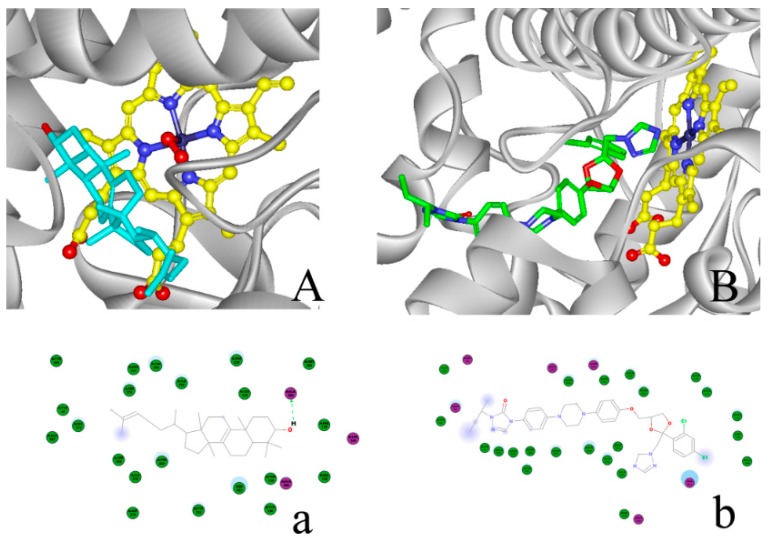
Lanosterol and itraconazole (ITC) binding in CYP51F1. (**A**) Lanosterol is depicted with carbon atoms colored cyan and heme with carbon atoms colored yellow. Selected oxygen atoms are colored red; nitrogen is blue; and iron is stone blue; (**B**) ITC is depicted with carbon atoms colored green, and other atoms are colored as in (**A**). A 2D diagram of the interaction between the protein and ligands is shown below: (**a**) lanosterol and (**b**) ITC. Purple dot: amino acids for a hydrogen bond, static electricity and polarity interactions. Green dot: amino acids for VDW (Van der Waals) interactions. Blue shadow: solvent around the amino acids and atoms. Green arrow: electron donor for a hydrogen bond.

The cavity volume at the binding sites was calculated, using DS (Binding Site module) for the CYP51F1 protein structure. A binding site with a maximum volume (x: 22.488, y: 8.09, z: 13.729; volume: 836.5) was selected manually, as it should have the appropriate volume for a molecule to adopt minimal energy. For monoterpenes (limonene, 3-carene and pinene) and sesquiterpenes (β-caryophyllene and longifolene), one pose of each was generated using “LibDock.” Seven DS scoring functions (Ligscore1, Ligscore2, -PLP1, -PLP2, Jain, -PMF and -PMF04) and consensus scoring functions were used to re-evaluate the position of docked molecules (Table 4). The position of terpenoid molecules were shown in the binding pocket of CYP51F1 colored green (Figure 6). The interactions of CYP51F1-terpenoid molecules were shown in a 2D diagram (Figure S1). For lanosterol and ITC, the most suitable docking mode for each molecule received a consensus score of seven and six, respectively. More than twelve amino acid residues participated in the interactions between CYP51F1 and the ligands lanosterol and ITC (Figure 7). Three (Ala 302, 306, Leu 303) and six (Ala 306, Met 70, His 373, Gly 69, Ser 374, Hem 601) amino acid residues formed hydrogen bonds, static electricity and polarity interactions between CYP51F1 and the ligands lanosterol and ITC, respectively (Figure 7). These sixteen amino acid residues (Ala 507, Val 135, Phe 130, 229, 234, Met 376, Thr 126, Leu 509, Tyr 68, 122, 136, 506, Pro 231, Ile 372, 375 and Ser 508) formed Van der Waals (VDW) interactions in both CYP51F1-lanosterol and CYP51F1-ITC (Figure S2).

**Table 4:** Seven DS scoring functions and consensus scoring function for terpenoid molecules and epoxiconazole.

Molecules	Ligscore1_Dreiding	Ligscore2_Dreiding	-PLP1	-PLP2	Jain	-PMF	-PMF04	Consensus Score
Limonene	−0.91	0.54	58.95	59.85	2.15	39.96	17.08	6
3-Carene	−0.69	0.93	53.39	54.82	1.66	42.01	19.76	4
Pinene	−0.66	0.96	44.28	47.31	3.36	20.88	6.1	3
β-Caryophyllene	−4.37	−5.11	61.84	64.42	3.77	41.38	3.55	4
Longifolene	−3.37	−3.54	57.63	62.2	4.62	36.29	7.93	4

### 2.5. MIC (Minimal Inhibitory Concentrations) of the Azole Fungicide and Monoterpenes

The MIC of ITC was 0.25 and 0.5 μg/mL for epoxiconazole (Table 5A). The monoterpenes could inhibit the spore germination with an increase in the concentration. With a concentration up to 200 μL/mL, over 90% of the spores were inhibited (Table 5B).

**Table 5:** Susceptibility pattern of tested *L. qinlingensis* to azole fungicide and monoterpenes. (**A**) Susceptibility pattern of tested *L. qinlingensis* to azole fungicide; (**B**) susceptibility pattern of tested *L. qinlingensis* to monoterpenes.

**(A)**																	
**Fungicide**	**Concentration (μg/mL)**																**Control**
	**4**	**2**		**1**		**1/2**		**1/4**		**1/8**		**1/16**		**1/32**		**0**	
ITC	−	−		−		−		−		+		+		+		+	−
	−	−		−		−		−		+		+		+		+	−
	−	−		−		−		−		+		+		+		+	−
	−	−		−		−		−		+		+		+		+	−
	−	−		−		−		−		+		+		+		+	−
Epoxiconazole	−	−		−		−		+		+		+		+		+	−
	−	−		−		−		+		+		+		+		+	−
	−	−		−		−		+		+		+		+		+	−
	−	−		−		−		+		+		+		+		+	−
	−	−		−		−		+		+		+		+		+	−
**(B)**																	
**Monoterpenes ^a^**			**Concentration (μL/mL)**												**Control**		
			**20**		**50**		**100**		**150**		**200**		**0**				
Number of Colonies			72		43		65		54		20		>150		>150		
			81		73		56		22		7		>150		>150		
			52		49		53		47		12		>150		>150		
Total			205		163		174		123		39		>450		>450		

^a^ The mixture of four monoterpenes ((+)-limonene (95%), (+)-3-carene (90%), (±)-α-pinene (98%) and (−)-β-pinene (99%) at a ratio of 5:3:1:1). −, inhibition; +, no inhibition.

## 3. Discussion

We report one fungal CYP51 gene of *L. qinlingensis* and its expression profile in mycelia treated with a terpenoid blend and pine phloem extracts. This gene is similar to variants of 14-α demethylase reported in *G. clavigera* kw1407 using genome and transcriptome analyses. Under experimental conditions, *L. qinlingensis* is similar to some other species through gene cloning and real-time fluorescent quantitative PCR [13]. Meanwhile, we also performed homology modeling of the structure of the CYP51F1 gene and molecule docking of some terpenoids, lanosterol and ITC.

In this study, we found that CYP51F1 partial and full-length amino acid sequences of *D. armandi* have sequence identity >75% with those of *G. clavigera*, *N. crassa*, *N. tetrasperma*, *M. thermophila*, *T. terrestris* and *O. piceae*. The lanosterol 14-α demethylase sequence from *G. clavigera* has the highest identity with 91.1% of the full-length sequence (Table 1). The ML-phylogenetic analysis of the putative full-length amino acid sequences shows that CYP51F1 has the highest homology with the lanosterol 14-α demethylase sequence from *G. clavigera*, as well (Figure 1). CYP51 is considered one of the most ancient families, which presumably evolved before the divergence of the major eukaryotic groups, and it exists in all biological kingdoms, but was lost in certain lineages, including insects and nematodes, that are heterotrophs with respect to sterols [27,28]. As *G. clavigera* is one of the major fungal associates of *D. ponderosae* [29], *L. qinlingensis* has some degree of homology with *G. clavigera*, as they are associates of two bark beetles in the *Dendroctonus* genus, even though they are in different genera. Except the two insect-dispersed ophiostomatoid species (*G. clavigera* and *O. piceae*), the CYP genes of many other pathogen species of ophiostomatoid, like Dutch elm disease pathogens *Ophiostoma ulmi* [30] and *O. novo-ulmi* [31], and *Ceratocystis moniliformis* and *C. manginecans* [32] were discovered with the genome sequence.

Multiple sequence alignments of these CYP51 genes to the *S. cerevisiae* lanosterol 14-α-demethylase protein sequence shows that they have similar regions, such as the heme-binding region (FXXGXRXCXG), PERF domain (PXRX) and K-helix (EXXR) (Figure 2) [33]. The likely substrate-binding site has been identified in CYP51F1 based on the analysis of multiple sequence alignment, some of which interact with azole inhibitors [34]. The theoretical analysis to infer the cellular localization of the deduced cytochrome P450 enzyme in *L. qinlingensis* indicates that it is most likely anchored to the outer face of the endoplasmic reticulum (Table 2). This inference is similar to that obtained from the homology modeling structure based on the crystal structure of the *S. cerevisiae* lanosterol 14-α-demethylase protein (Figure 5).

Previous studies have suggested that CYP51 proteins have a preserved narrow function of removing the 14-methyl group of sterol precursors and have retained high substrate specificity throughout evolution [17]. For fungus, we always focus on two aspects of CYP51 genes: the biosynthesis of ergosterol, a sterol specifically found in fungal membranes that mediates their permeability and fluidity [35], and azoles that interfere with fungal lanosterol 14-α-demethylase to affect the function of essential membrane-bound enzymes [36,37,38,39].

To colonize pine trees, *L. qinlingensis* must cope with host defense chemicals, including terpenoids and phenolics, that are toxic to many fungal species. The pathogenic fungus of pine trees must retrieve nutrients, primarily carbon, from its host by accessing sugars, triglycerides and organic nitrogen, to develop its mycelia and reproductive structures [14]. Pine defense chemicals induced abundant transcription of many genes of *G. clavigera*, including CYP genes, changed significantly following exposure to either a complex terpenoid blend or lodgepole pine extract (LPPE) containing phenolics and other metabolites [13,26].

CYP51F1 transcripts overexpressed at 12 and 36 h in mycelia, after treatment with a terpenoid blend and CWPPE, had similar transcript levels in untreated mycelia (Figure 3). The terpenoid blend was more influential than CWPPE. The significant downregulation of CYP51F1 due to the monoterpene blend as the single carbon source also suggests that terpenoid metabolism may be connected with CYP51F1.

Molecule docking of monoterpenes (limonene, carene and pinene) and sesquiterpenes (β-caryophyllene and longifolene) shows that small terpenoid molecules can occupy the binding pocket of CYP51F1 (Figure 6). Almost every amino acid residue that is involved in the VDW interactions of CYP51F1-terpenoid molecules (Figure S1) is involved in the interactions of CYP51F1-lanosterol. The antifungal triazole drug ITC extends from the active site to just beyond the mouth of the entry channel, similar to ITC in the *S. cerevisiae* and posaconazole in the *T. brucei* CYP51 structure [40,41]. The space occupied by ITC (Figure 7B) fits closely with that occupied by lanosterol and O_2_ (Figure 7A), with the triazole head group displacing the O_2_ and the di-halogenated headgroup replacing the first sterol ring. The MIC determination of the azole fungicides and monoterpenes suggests that they can inhibit the reproduction of *L. qinlingensis*.

In brief, the results of this research provide important information suggesting that terpenoids from the host tree *P. armandi* impede the CYP51F1 enzyme from playing a role in lanosterol oxidation. The action of these terpenoids to inhibit *L. qinlingensis* is similar to azole fungicides.

## 4. Experimental Section

### 4.1. Strains

*Leptographium qinlingensis* (NCBI Taxonomy ID: 717526) was deposited at the College of Forestry, Northwest A&F University (Yangling, China).

### 4.2. Fungal Media and Growth Conditions

*L. qinlingensis* was grown on the medium containing 0.83% Oxoid malt extract agar and 0.75% technical agar (Oxoid Ltd., Basingstoke, Hampshire, UK) overlaid with cellophane, and the pH was adjusted to 5–6. Mycelia used for extracting RNA were collected from solid media inoculated with a suspension containing 5 × 10^5^ spores and were incubated for 5–7 days (depending on the library) at 28 °C in the dark.

### 4.3. Obtaining the CYP51 Gene

#### 4.3.1. RNA Isolation and cDNA Synthesis

Total RNA was isolated from mycelia according to the protocol supplied with the E.Z.N.A™ Fungal RNA Kit (Omega Bio-Tek, Norcross, GA, USA). Its integrity was assessed on 1% agarose gels, and quantification was performed by spectrophotometry with a NanoDrop 2000 (Thermo Scientific, Pittsburgh, PA, USA). The purity was estimated by the means of the A260/A280 equation (μg/mL = A260 × dilution factor × 40). The cDNA was synthesized using the EasyScript™ First-Stand cDNA Synthesis SuperMix (TransGen Biotech, Beijing, China) according to the manufacturer’s instructions.

#### 4.3.2. Amplification of Genes, Cloning and Sequence Analyses

The synthesized cDNA obtained from the sample was used as a template in PCR reactions. A pair of degenerate primers was designed to screen the putative P450 cDNA from the CYP51 family (Table S1).

PCR amplifications were performed in a C1000 thermocycler (Bio-Rad, Hercules, CA, USA). CYP51 genes were amplified under the indicated conditions in 20-μL reactions containing 1 μL cDNA, 0.25 μM of each primer and 1× EcoTaq PCR SuperMix (TransGen Biotech, Beijing, China). An initial 5-min step at 94 °C was followed by 30 cycles of 30 s at 94 °C, 30 s at 41 °C and 30 s at 72 °C, with a final extension for 10 min at 72 °C.

The PCR products were visualized on 1% agarose gels stained with 1× DuRed and compared with a 2K plus DNA marker (TransGen Biotech, Beijing, China). Amplicons were purified using the Gel Purification Kit (Spin-column) (Bio Teke, Beijing, China), and the reaction product was cloned using the pMD™ 18-T Vector (TaKaRa, Dalian, China).

Cloning reactions were transformed into DH5α chemically-competent cells of *Escherichia coli*, and the transformants (blue-white colonies) were selected on Amp/LB/X-gal/IPTG plates. A total of 10 clones with inserts were sequenced directly by GenScript USA Inc. (Nanjing, China).

The sequences were manually edited with DNAMAN to obtain the insert sequences. Blastx searches of partial-length sequences (approximately 500 bp) were made against the NCBI database. The sequences were translated into amino acid sequences with the ExPASy Translate Tool (http://www.expasy.org/tools/dna.html) and subjected to a BlastP search against the GenBank database [23]. A multiple sequence alignment of the P450 proteins was performed with ClustalX v2.0.10 using default parameters [42].

#### 4.3.3. End Sequence Determination and Cloning of Full-Length cDNAs

The complete sequence of the CYP51 gene identified above was achieved using the SMARTer™ RACE cDNA Amplification Kit (Clontech Laboratories Inc., Mountain, CA, USA). The total RNA of mycelia was obtained, following the protocol described in the E.Z.N.A™ Fungal RNA Kit (Omega Bio-Tek, Norcross, GA, USA); its integrity was assessed on 1% agarose gels.

Partial sequences were used in the primer design, and PCR was performed following the protocol described in the SMARTer™ RACE cDNA Amplification Kit (Clontech Laboratories Inc., Mountain, CA, USA). The amplicons were purified, cloned and sequenced as previously described. The complete sequences were compared using a BlastP search with those deposited in GenBank [23].

To avoid chimera sequences, we designed specific primers (Table S1) based on the complete sequence obtained for the CYP51 gene with RACE; the specific primers were used to amplify the complete DNA for each gene. Amplification reactions were carried out in 20-μL volumes containing: 1 μL cDNA from a 1:5 dilution, 0.25 μM of each primer and 1× EcoTaq PCR SuperMix (Beijing TransGen Biotech Co., Ltd., Beijing, China). The PCR reactions were performed as follows: 94 °C for 5 min, 30 cycles of 94 °C for 30 s, 68 °C for 30 s and 72 °C for 2 min, with a final extension for 10 min at 72 °C.

PCR products of approximately 2000 bp were visualized on 1% agarose gels, purified and cloned, and both strands were sequenced as previously described. The deduced amino acid sequences were submitted to the P450 nomenclature committee, and a name was assigned based on their criteria for the classification of CYP51 genes (David Nelson Department of Molecular Sciences, University of Tennessee, personal communication). The sequence was deposited in GenBank (Accession Number KJ569144).

#### 4.3.4. Analysis of the Full-Length Cytochrome P450 Sequence

To identify the different CYP variants expressed in fungus, a phylogenetic inference analysis by maximum likelihood of the full-length CYP sequence was performed with MEGA5 [43]. CYP topology was used to identify groups, but not to establish a phylogenetic relationship. The WAG model was supported by the test (−lnL = −2883.910) with a gamma parameter value of G = 0.39. To estimate the support of each node, bootstrap values were calculated after 500 pseudoreplicates. *Neurospora crassa* OR74A (cytochrome P450 51, Accession Number EAA34813.2), *Neurospora tetrasperma* FGSC 2509 (cytochrome P450 51, Accession Number EGZ77871.1) *Myceliophthora thermophila* ATCC 42464 (eburicol 14-α demethylase, Accession Number AEO53391.1), *Thielavia terrestris* NRRL 8126 (eburicol 14-α demethylase, Accession Number AEO70267.1) and *Ophiostoma piceae* UAMH 11346 (cytochrome p450 51b, Accession Number EPE08931.1) were used as the outgroup sequences in the analysis. A multiple sequence alignment of the P450 proteins was performed with ClustalX v2.0.10 using default parameters [42]. Except for the sequences above, lanosterol 14-α demethylase of *S. cerevisiae* (PBD code: 4k0f, chain A) was used to determine the substrate recognition sites (SRS).

Finally, MATGAT v2.01 software was used to determine the identity percentages among partial-length amino acid sequences, full-length sequences and GenBank sequences from other fungi (interspecific identity) [22].

The molecular mass (kDa) and isoelectric point (pI) of the sequence were determined using the ProtParam program [24]. All putatively functional *L. qinlingensis* P450 proteins were examined for likely sub-cellular localization using the TargetP program (http://www.cbs.dtu.dk/services/TargetP/) with the default parameters [25].

### 4.4. Real-Time Fluorescent Quantitative PCR

#### 4.4.1. Treatments for RT-qPCR

We generated and analyzed transcript level data from two sets of growth conditions. For the first set of conditions, mycelia were generated from a suspension of 5 × 10^5^ spores spread on cellophane on the surface of complete media (CM: 0.17% yeast nitrogen base without amino acids (YNB; BD Difco, Sparks, MD, USA), 1.5% agar, 1% maltose, 0.1% phthalate, 0.3% asparagine). The spores were grown for 3 days at room temperature before being treated with either a crude Chinese white pine phloem methanol extract (CWPPE; CM + CW) or with a terpenoid blend (CM + T) and further incubated for 12 and 36 h. The CWPPE was prepared using DiGuistini’s method for preparing the lodgepole pine phloem methanol extract (LPPE) [13]. The CWPPE contained methanol-soluble phenolic chemicals, sugars and possibly other metabolites, while the complex terpenoid blend included monoterpenes ((+)-limonene, (±)-α-pinene, (−)-β-pinene, (+)-3-carene) and turpentine (mainly consists of terpenes). The relevant controls for CM + CW and CM + T treatments were mycelia grown on CM with methanol and CM, respectively.

In the second set of conditions, we tested the utilization of different carbon sources by *L. qinlingensis*. Again, the mycelia were generated from spores grown on 1% MEA (0.83% malt extract agar and 0.75% technical agar (BD Difco, Sparks, MD, USA )) overlaid with cellophane for 3 days. The young germinating mycelia were transferred to minimal media (YNB: 1.5% agar with 0.67% yeast nitrogen base without amino acids; BD Difco, Sparks, MD, USA) with either a mixture of monoterpenes (YNB + MT; (+)-limonene (95%), (+)-3-carene (90%), (±)-α-pinene (98%) and (−)-β-pinene (99%) at a ratio of 5:3:1:1), 0.5% oleic acid (YNB + OA) or 1% mannose (YNB + Ma). While the oleic acid and mannose were incorporated into the media, the monoterpene mixture (200 μL) was sprayed onto the surface of the media. Mycelia on oleic acid were incubated for 5 days, while mycelia with monoterpenes were incubated for 10 days. We used mycelia grown on YNB + Ma for 3 days as a control for YNB + MT and mycelia grown on YNB + Ma for 5 days as a control for YNB + OA. These time points were chosen when the mycelial growth approximately reached confluence for the treatment and control conditions. Monoterpenes were from Sigma Aldrich (St. Louis, MO, USA), and other chemicals were analytically pure and made in China.

#### 4.4.2. RNA Isolation and cDNA Synthesis for Expression Analyses

The total RNA isolation of the fungi was performed following the protocol described in the E.Z.N.A™ Fungal RNA Kit (Omega Bio-Tek, Norcross, GA, USA), and its integrity was verified in 1% agarose gels. The cDNA synthesis was performed using the protocol described in the FastQuant RT Kit (with gDNase) (Tiangen Biotech Co., Beijing, China) using 2 μg total RNA in a 20-μL final reaction volume. The cDNA synthesis program was as follows: 42 °C for 15 min and 95 °C for 3 min. A non-reverse transcription assay was performed to evaluate the DNA absence in the RNA extraction. The cDNA was stored at −20 °C.

#### 4.4.3. RT-qPCR

For each target gene and reference gene, specific primers were designed using Primer Premier 5.0 (Table S1). The reaction was carried out under the following conditions: each PCR reaction contained 0.4 μM of each primer, 12.5 μL FastStart Essential DNA Green Master (Roche Diagnostics GmbH, Mannheim, Germany) and 2 μL of the diluted cDNA sample in a final volume of 25 μL. All of the samples were place in the CFX96™ Real-Time PCR Detection System (Bio-Rad, Hercules, CA, USA). A 3-step amplification condition with a hot-start step was used: 95 °C for 10 min, 95 °C for 30 s and 40 cycles at 95 °C for 5 s, 60 °C for 30 s and 72 °C for 30 s. PCR contaminations were not detected in the no template control (NTC). The experiment was replicated three times (biological replicates), and each of the replicates was performed with three technical replications.

To estimate the qPCR efficiency and validation for each gene, a linear regression analysis was performed between the mean values of the quantification cycles (Cq) of different dilutions (1.0, 10^−1^, 10^−2^, 10^−3^, 10^−4^) of cDNAs and the initial concentration. These dilutions were made from a cDNA pool, and 2 μL of each dilution were used as a qPCR template. The PCR was performed three times for each gene, and its efficiency was estimated with the equation: efficiency = (10^−1/slope^ − 1) × 100, where the *E* value was 100% ± 5%. The PCR validation was estimated directly from the *R*^2^ values, which were >0.90. Moreover, a melting curve reaction was performed to evaluate their specificity.

#### 4.4.4. Reference Gene Validation Experiment

We used the same method to generate three gene partial sequences (28s rRNA, EF1 and calmodulin) as reference genes. The obtained three gene sequences were deposited in GenBank (Accession Number KJ541045-KJ541047). Calculations to estimate the expression stability were performed with the geNorm program [44]. EF1 was the most stable gene, so the expression levels of the gene were normalized to the expression levels of EF1.

#### 4.4.5. Statistical Analysis

Relative expression values for all of the genes were determined using the *C*_t_ (ΔΔ*C*_t_) method and analyzed with Microsoft Excel 2003 (v.11.0.5612) [45]. Outlier values identified by a PCR machine were excluded from our analysis. To evaluate significant differences in the expression for each gene, 2^−ΔΔ*C*t^ values transformed at log_2_ were subjected to one-way ANOVA to determine if the gene expression was different among the treatments. The 2^−ΔΔ*C*t^ values and standard error (SE) were transformed at log_2_ to generate graphs. All of the statistical analyses were performed with SPSS 18.0 (IBM SPSS Statistics, Chicago, IL, USA) and plotted with SigmaPlot 12.0 software (Systat Software Inc., San Jose, CA, USA).

### 4.5. Protein Structure and Ligand Docking

#### 4.5.1. Homology Modeling

Four proteins (*S. cerevisiae* YJM789 lanosterol 14-α demethylase PDB code: 4k0f.1 chain A, 4lxj.1 chain A, *Homo sapiens* lanosterol 14-α demethylase PDB code: 3ld6.1 chain B, 3juv.1 chain A) (identity > 35%) were selected from the SWISS-MODEL Repository (http://swissmodel.expasy.org/repository/) as suitable templates. Homology modeling was performed with the SWISS-MODEL program for the CYP51F1 protein [46]. The four modeled protein structures were verified using the Structural Analysis and Verification Server (SAVES) (http://nihserver.mbi.ucla.edu/SAVES/), which uses different programs, such as ERRAT to evaluate the statistics of non-bonded interactions between different atom types [47], VERIFY 3D to determine the compatibility of the 3D atomic model with its own amino acid sequence and PROCHECK to assess the stereo chemical quality of a protein structure by analyzing residue-by-residue geometry and overall structural geometry [48,49].

#### 4.5.2. Protein-Ligand Interaction Study

Discovery Studio v2.5 (DS 2.5) (Accelrys, San Diego, CA, USA) was used to dock monoterpenes (limonene, 3-carene and pinene), sesquiterpenes (β-caryophyllene and longifolene), lanosterol and itraconazole (ITC) to our refined model. Two of the preferred ligands were lanosterol and ITC. The terpenoid molecules were built using “Molecular Window” and optimized using “Prepare Ligands” in the DS for docking. The lanosterol and ITC molecule structures were retrieved from DrugBank 4.0 (http://www.drugbank.ca/) with Accession Numbers DB01167 and DB03696. “LibDock” and “LigandFit” in the DS were used to dock ligand molecules into the refined model [50,51]. The terpenoid molecules were docked into the refined model using only “LibDock.” For lanosterol and ITC, 10 poses of each were generated using “LigandFit” and scored using the DS scoring functions, which include Ligscore1, Ligscore2, -PLP1, -PLP2, Jain and -PMF. Among these poses, the most suitable docking mode for each molecule with a high score from the consensus scoring functions was finally selected. Furthermore, protein-ligand interactions of all of the molecules were shown in a 2D diagram.

### 4.6. Determination of the MIC of Terpenoid and Azole Fungicides

The CYP51 inhibitors ITC and epoxiconazole were selected as representative agricultural azoles for a MIC screening. ITC and epoxiconazole were dissolved in dimethyl sulfoxide (DMSO) to obtain stock solutions of 1600 μg/mL. All drugs were stored at −20 °C. A 1% malt extract microdilution susceptibility assay was performed according to the Clinical and Laboratory Standards Institute M38-A2 protocol in order to evaluate the initial MIC of ITC and epoxiconazole. The final drug concentration ranged from 0.03125–4 μg/mL for both ITC and epoxiconazole. An equal volume of 1 × 10^5^ spores was mixed with the 1% malt extract microdilution susceptibility assay. The MIC of azoles was defined as the lowest concentration of the drug that produced no visible growth following 72 h of incubation at 27 °C. The MIC determination was repeated five times.

The mixture of monoterpenes ((+)-limonene (95%), (+)-3-carene (90%), (±)-α-pinene (98%) and (−)-β-pinene (99%) at a ratio of 5:3:1:1) was dissolved in 0.01% Tween 80 to obtain 40-, 100-, 300- and 400-μL/mL emulsions. An equal volume of 5 × 10^4^ spores was mixed with the emulsion to bring the final monoterpenes concentration range from 20 to 200 μL/mL. In total, 20 μL of the mixture was spread on the 1% MEA to count the number of colonies after 48 h of incubation at 27 °C. A mixture with 0.01% Tween 80 and sterile water was spread on the 1% MEA as the control. Each concentration and the control were repeated three times.

## 5. Conclusions

The transcription level of CYP51F1 following treatment with terpenes and pine phloem extracts was upregulated, while using monoterpenes as the only carbon source led to the downregulation of CYP5F1 expression. The homology modeling structure of CYP51F1 is similar to the structure of the lanosterol 14-α demethylase protein of *Saccharomyces cerevisiae* YJM789, which has an *N*-terminal membrane helix 1 (MH1) and transmembrane helix 1 (TMH1). The minimal inhibitory concentrations (MIC) of terpenoid and azole fungicides (itraconazole (ITC)) and the docking of terpenoid molecules, lanosterol and ITC in the protein structure suggested that CYP51F1 may be inhibited by terpenoid molecules by competitive binding with azole fungicides.

## Supplementary Materials

Supplementary materials can be found at http://www.mdpi.com/1422-0067/16/06/12014/s1.
