# Supplementary material for: The CYP51F1 Gene of Leptographium qinlingensis: Sequence Characteristic, Phylogeny and Transcript Levels
Source: Int J Mol Sci. 2015 May 26;16(6):12014–34. doi: 10.3390/ijms160612014 (PMC4490426; doi:10.3390/ijms160612014)
Supplement: Supplementary file 1 [file ijms-16-12014-s001.pdf]

Supplementary Information

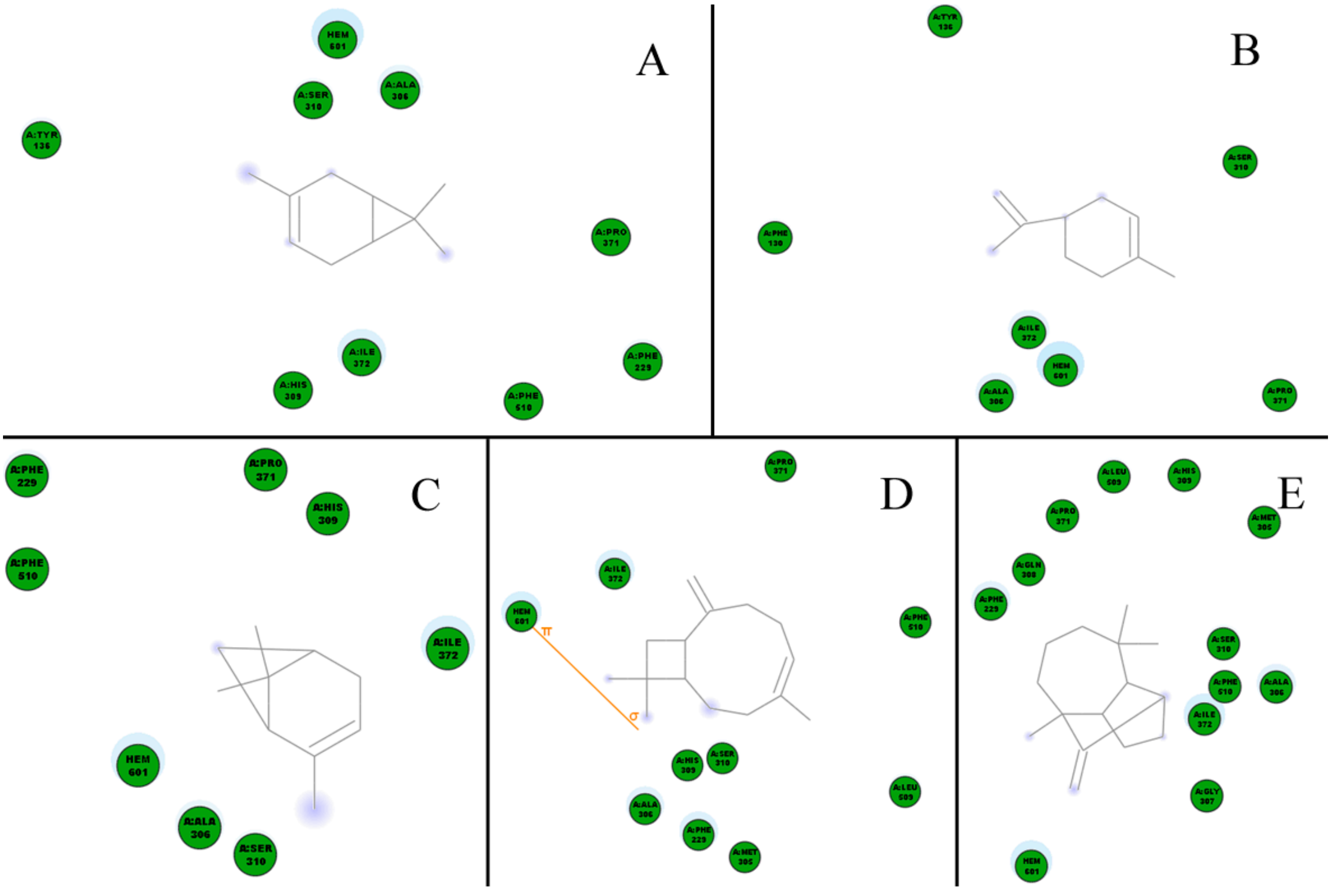

Figure S1. 2D diagram of the interaction between protein and ligands showed: limonene (**A**); carene (**B**); pinene (**C**); β-caryophyllene (**D**) and longifolene (**E**). Green dot: amino acids for VDW interactions. Blue shadow: solvent around the amino acids and atoms. Orange line: Pi-Sigma interaction.

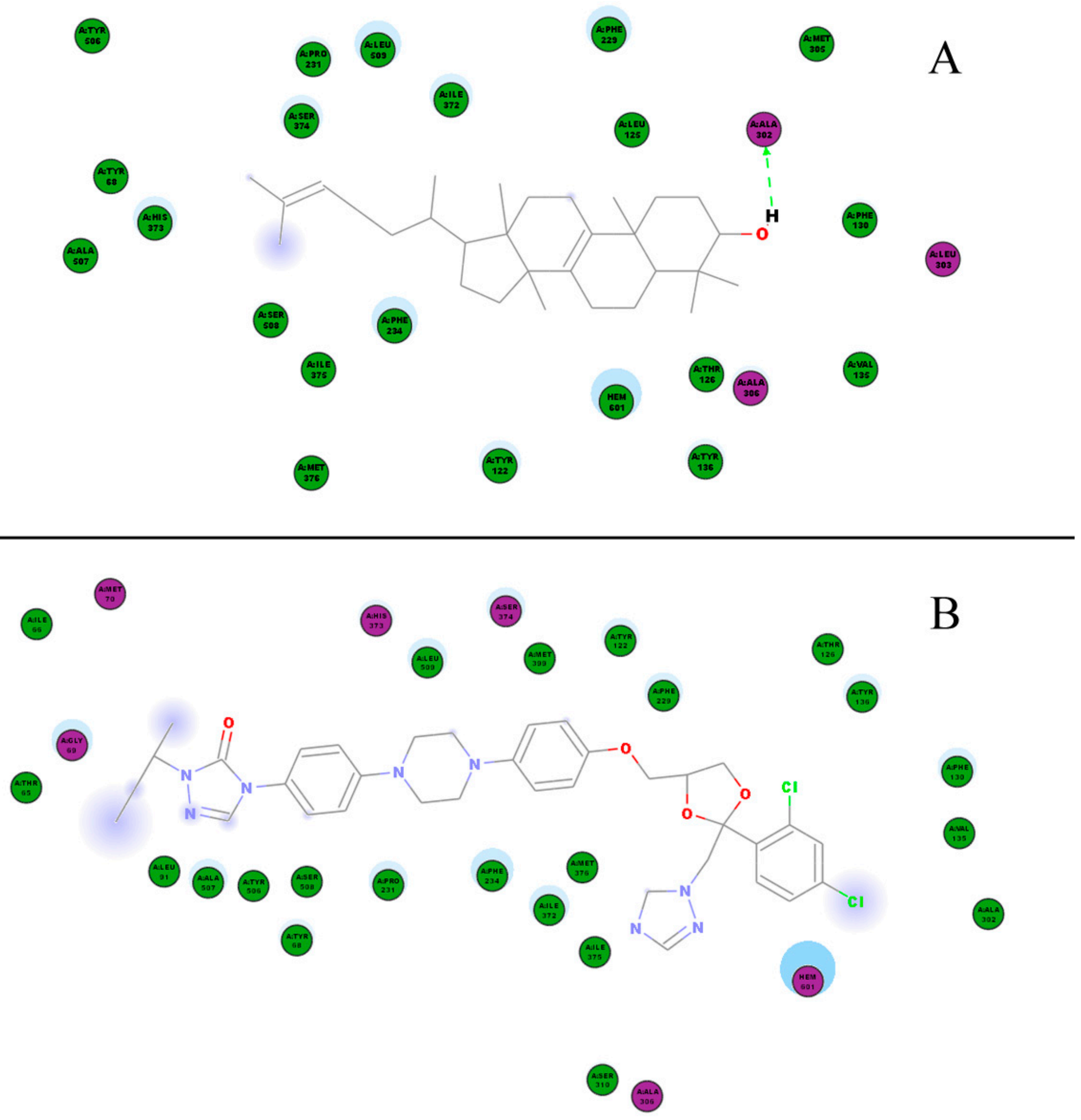

Figure S2. 2D diagram of the interaction between protein and ligands showed: (**A**) lanisterol and (**B**) ITC. Purple dot: amino acids for hydrogen bond, static electricity and polarity interactions. Green dot: amino acids for VDW interactions. Blue shadow: solvent around the amino acids and atoms. Green arrow: electron donor for hydrogen bond.

Table S1. Primers used in this study.

Gene	Primer	Sequence(5′–3′)
CYP51	Degenerate	F- TYATGGAGCAGAAGAAGTT<br>R- RAGRAGVGCRATCATCAT
CYP51F1	3′RACE	GSP- CATCATCTCGGAAGAGGTCAACAGC<br>NGSP- GGAACCAAGTATGTTGTGCCTACCAGC
	5′RACE	GSP- AGCCCATGTCCAGGTCGTGGTAC<br>NGSP- AAACTGGTTGCGGACCTCCTTGC
	Full-length validation	F- AGCCGCGTGGACAGACACTTG<br>R- GATTAAAAGGCGTGTACGGATTA
	RT-qPCR	F- TTCAAGAACCCGCACGAGCCA<br>R- GCCGAGCAGGACGAAAGAGTA
28s rRNA	Amplification	F- AGCGAACTGCGATAAGTAATG<br>R- TTCACAAAGGCTAGGTGTCC
	RT-qPCR	F- AGCTGTTGCCGCTTCACTCG<br>R- GGTTGACCTCGGATCAGGTAGG
EF1	Amplification	F- GCTGCTGTCCGTGTTGAA<br>R- GGTTGTAGCCGACCTTCTT
	RT-qPCR	F- CTTGGTGGTGTCCATCTTGTT<br>R- CCGCTGGTACGGGTGAGTT
Calmodulin	Amplification	F- CAGGCCAAATCACCACCA<br>R- TCCTCGTCCGTCAGCTTCT
	RT-qPCR	F- CCAAATCACCACCAAGGAGC<br>R- GTGCCATCATCGTGAGGAACT

F = forward primer, R = reverse primer; GSP = Gene Special Primer, NGSP = Nest Gene Special Primer.
